# Neutrophil extracellular traps drive peritoneal inflammation and tissue remodeling in pediatric peritoneal dialysis

**DOI:** 10.1007/s00467-025-07003-w

**Published:** 2025-10-18

**Authors:** Charlotte Maria Dücker, Martin Herrmann, Susanne Boettcher, Sarah Bauer-Carmona, Raphael-Sebastian Schild, Lavinia Schönfeld, Laia Pagerols Raluy, Konrad Reinshagen, Claus Peter Schmitt, Maria Bartosova Medvid, Michael Boettcher

**Affiliations:** 1 Department of Pediatric Surgery, University Medical Center Hamburg-Eppendorf, Hamburg, Germany; 2 Department of Pediatric Surgery, University Medical Center Mannheim, University of Heidelberg, Mannheim, Germany; 3 Department of Pediatrics, University Medical Center Hamburg-Eppendorf, Hamburg, Germany; 4 Institute of Human Genetics, University Hospital Heidelberg, Heidelberg, Germany; 5 Heidelberg University, Medical Faculty Heidelberg, Centre for Pediatrics and Adolescent Medicine, Clinic 1, Pediatric Nephrology, Heidelberg, Germany

**Keywords:** Peritoneal dialysis, Neutrophil extracellular traps, DNase1 and DNase1L3, Children

## Abstract

**Background:**

Peritoneal dialysis (PD) sustains children with chronic kidney disease stage 5 (CKD5) but promotes peritoneal membrane remodeling. Neutrophil extracellular traps (NETs) orchestrate antimicrobial defense and sterile inflammation; their involvement in PD-induced transformation is unknown.

**Methods:**

Forty-five children were enrolled in the International Pediatric Peritoneal Biobank. Peritoneal biopsies taken at PD initiation and after ≥ 12 months of low-glucose-degradation-product PD were compared with surgical biopsies from non-uremic peers. Histomorphometry quantified microvessel density, submesothelial thickness, leukocyte infiltration, collagen I/III, and NET markers (citrullinated histone H3, neutrophil elastase, myeloperoxidase). Dialysate and plasma collected every 2 months for 18 months were assayed for cell-free DNA, NET proteins, DNase1, and DNase1L3.

**Results:**

After chronic PD, the peritoneum displayed doubled microvessel density, tripled submesothelial thickness, and marked immune-cell infiltration (all *p* < 0.01). NET structures were prominent in tissue, while dialysate and plasma concentrations of cell-free DNA, citrullinated histone H3, neutrophil elastase, and myeloperoxidase increased two- to fourfold versus baseline (*p* < 0.05). DNase1 levels correlated with membrane thickness (*r* = 0.46, *p* = 0.003) and DNase1L3 with vascular density (*r* = 0.51, *p* = 0.001), suggesting limited compensatory NET clearance.

**Conclusions:**

Chronic PD elicits NET-driven sterile inflammation that parallels structural remodeling of the pediatric peritoneum. Supplementing PD fluids with exogenous NET-degrading enzymes may preserve membrane integrity and prolong PD suitability in children.

**Graphical Abstract:**

**Figure:**
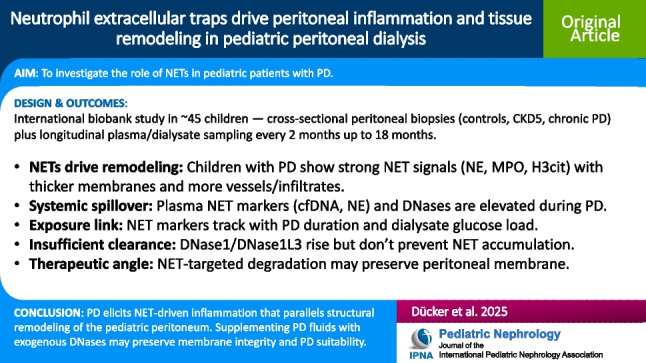
A higher resolution version of the Graphical abstract is available as Supplementary information

**Supplementary Information:**

The online version contains supplementary material available at 10.1007/s00467-025-07003-w.

## Background

Peritoneal dialysis (PD) is the preferred kidney replacement therapy in children with chronic kidney disease (CKD5) when transplantation is not an option. A semipermeable peritoneal membrane serves for removal of toxins and excessive water from the patient. To achieve the osmotic gradient, high concentrations of glucose are instilled into the peritoneal cavity acting on the peritoneal membrane [1, 2]. A major limitation of long-term PD is the peritoneal remodeling with alterations of the membrane structure and function over time, resulting in significant reduction of PD effectiveness. Several studies in vivo and in vitro showed that single chamber fluids containing glucose, lactate, and high concentrations of glucose degradation products (GDP) induce damage of the mesothelial cells lining the peritoneum, causing peritoneal membrane thickening, and vascular disease [3–5]. Moreover, GDP are taken up systemically, react with proteins forming the advanced glycation products which deposit on endothelium of the vessel and are associated with major vascular disease. The double-chamber PD fluids contain far less GDP, but still high concentrations of glucose. In clinical trials, usage of low GDP PD fluids preserved residual kidney function and was associated with fewer episodes of peritoneal infection, peritonitis [6–8]. While high GDP PD fluids induce apoptosis and cell death [3, 9], low GDP PD fluids are associated with sterile inflammation [10–12]. This induced remodeling of peritoneal tissue leads to PD-associated complications which could result in PD failure. Peritoneal tissues from children treated with low GDP PD fluids exhibit infiltration with immune cells and major vascularization as soon as 4 months after initiation of PD [11, 13]. These morphological changes are reflected in clinical parameters: patients receiving low GDP PD showed an initially higher D/P ratio, which remained stable over time [6, 8].

Despite the severe clinical consequences of peritoneal membrane remodeling, the underlying pathophysiology is still elusive. Evidence from omics analysis and tissue studies suggests that inflammation is a major driver of peritoneal transformation caused by high glucose levels in PD fluids [14, 15]. The innate immune system reacts immediately through a plethora of tightly regulated mechanisms endowed with both pro- and anti-inflammatory activities that last for several days [16].

In response to infection or sterile tissue injury, neutrophils form NETs, which consist of bundles of high molecular weight double-stranded DNA bundles that build robust scaffolds [17–19]. NETs are multi-functional structures that regulate both homeostatic and pathological inflammation [20, 21]. During infection NETs exhibit antimicrobial functions, and trap, immobilize, and eventually kill extracellular pathogens in blood and tissue [22, 23]. However, NETs also form during sterile inflammation, stimulating platelet adhesion and coagulation [24]. While the function of NETs is essential in combating infection and inflammatory responses, a spatial and temporal inappropriate production of NETs can have detrimental effects. NETs contribute to the pathology of several inflammatory conditions, such as autoimmune diseases, sepsis, and ischemia reperfusion injury of intestine and testicle [25, 26]. Recently, it has been reported that they form the scaffold leading to adhesion formation [27–29].

We argue that excessive formation of NETs and aggregated NETs represent an important pathomechanism in dialysis-induced inflammation observed in peritoneal tissues of children exposed to high glucose content in dialysate, and we analyzed the local peritoneal and systemic formation of NET markers (cfDNA, H3cit, NE, MPO), DNase1, DNase1L3 as well as the transformation of peritoneal membranes and vessels.

## Materials and methods

### Study population

Peritoneal tissues were collected within The International Pediatric Peritoneal Biobank [11, 30]. The study was registered at www.clinicaltrials.gov (NCT01893710). It is in accordance with the guidelines of the medical research ethics committee of Hamburg (Ethik-Kommission der Ärztekammer Hamburg, MC-227/12), and with the 1964 Helsinki declaration and its later amendments. Written informed consent was obtained from the legal representatives. Parietal peritoneal tissues were fixed in 4% formaldehyde on cork plates and embedded in paraffin.

(1) 16 children without kidney disease (controls), (2) 12 children with chronic kidney disease stage 5 at the time of PD catheter insertion (CKD5 group) and (3) 17 children on chronic PD (PD group) with low GDP PD fluids were studied. Children with CKD5 and on PD suffered from congenital malformation of kidney and urinary tract (*n* = 23), congenital nephrotic syndrome (*n* = 2), nephronophthisis (*n* = 2), renal tubular dysgenesis (*n* = 1), and nephropathic cystinosis (*n* = 1). Tissues were collected during catheter insertion surgery or during kidney transplantation. The sampling site was at least 5 cm distant from the PD catheter entry site. All children had a BMI lower than the 85th age-related percentile.

### Blood and dialysate sampling

In all children without kidney disease, parallel to tissue samples, plasma samples were also collected. In 9 CKD 5 and 7 PD patients, plasma and dialysate samples were collected at the time of tissue collection and then prospectively every 2 months in a standardized way during routine clinical visits. Dialysate was collected after the overnight dialysis dwell, and dialysate and blood samples were stored in aliquots at −80 °C until further use and were used undiluted to avoid freeze–thaw cycles.

### Systemic NET formation and degradation markers

We quantified cell-free DNA with Sytox orange Nucleic Acid Stain (Thermo Fisher S11368, Waltham, MA, USA) and used a standard curve with serial dilution of lambda-DNA (Thermo Fisher SD0011, Waltham, MA, USA). Dilutions were prepared in 96-well plates with v-bottom (Sarstedt 821.583, Nuembrecht, Germany) and transferred to black flat bottom plates (Greiner 655076, Kremsmuenster, Austria) for fluorescence measurement. Citrullinated Histone H3 (H3cit) was quantified with an ELISA kit (Clone 11D3; Cayman chemical 501620, Ann Arbor, MI, USA). The TMB and HRP color reaction was stopped with acid. We measured human PMN-Elastase with an ELISA Kit (Thermo fisher, Invitrogen, BMS269, Waltham, MA, USA). For dialysate samples, the standard curve was performed with unused dialysis fluid to account for minor matrix effect. For human myeloperoxidase, we employed the human myeloperoxidase Quantikine ELISA (R&D Systems, DMYE00B, Minneapolis, MN, USA). All blood and dialysate fluid assay results were measured with fluorescence or optical density at a Flex Station 3 (Molecular Devices, San Jose, CA 95134, USA).

DNase1 was quantified with DNAse1 Quantification ELISA Kit (MyBiosource, MBS2515385, San Diego, CA, USA) and detected cell death with a detection ELISA Plus Kit (Roche, 11,920,685,001, Basel, Switzerland). For standard curve pooled samples were used.

### Immunostaining and digital histology

For histological analyses, 3 µm sections were used, two sections per slide and one serving as negative control (incubated with corresponding isotype control of primary antibody). For immunostaining (immunohistochemistry and immunofluorescence), the sections were deparaffinized and heat induced antigen retrieval was performed.

After washing with PBS and blocking, the following antibodies were used for DNase1: bs-7651R, 1: 100, Rabbit, Bioss Antibodies; Iso DNase1: ab37415, 1:500, Rabbit, Abcam and for DNase1L3: bs-7653R, 1:100, Rabbit, Bioss Antibodies; Iso DNase1L3: ab37415, 1:500, Rabbit, Abcam; NE: ab 68,672, 1:200, Rabbit, Abcam; Iso NE: ab 37,415, 1:2000, Rabbit, Abcam; MPO: ab25989, 1:50, Mouse, Abcam; Iso MPO: DAK-GO1, 1:5, Agilent; H3cit: ab 5103, 1:100, Rabbit, Abcam, Iso H3cit: ab 37,415, 1:300, Abcam.

After incubation with biotinylated secondary antibody (AF 647, Donkey anti Rabbit, 1:200, ab150075, Abcam; AF 647 Donkey anti Mouse, 1:200, 715–607-003, Jackson Imm Research; Cy3, Donkey anti Rabbit, 1:200, 715–165-152, Jackson Imm Research) against the host species of the first antibody for 30 min at RT, ABC reagent was applied (both Vector Laboratories, Newark, CA, USA). DAB + (Agilent, Waldbronn, Germany) was used for detection. Cell nuclei were counterstained with DAPI (Thermo Fisher Scientific, Waltham, MA, USA). For each antibody, an adapted isotype control antibody was used as a negative control.

To differentiate collagen I from III, we stained collagen fibers using Pico Sirius red (ab150681, Abcam) and evaluated them with a polarized light microscopy.

Histological sections were automatically imaged at 20 × magnification (resolution, 0,46 μm/pixel) using Hamamatsu NanoZoomer 2.0-HT Scan System (Hamamatsu Photonics, Hamamatsu, Japan). All stainings were digitally analyzed as established previously [11, 27]. Submesothelial thickness and submesothelial microvessel density were measured after CD31 staining (JC70A, M0823, 1:25, Agilent). Vasculopathy was analyzed as the ratio of luminal diameter to vessel external diameter (L/V). Arterioles of 60–100 µm diameter (5–7 per section) were analyzed. CD45 positive leukocyte (2B11 + PD7/26, 1:100, M0701, Aglient) and CD68 macrophage infiltration (PG-M1, 1:100, M0876) were quantified per cell count per HPF. The assessment of collagen alignment was scored based on the orientation of the bundles (1, diffuse with bundles in 90°/perfectly random; 2, mostly bundles, random; 3, both, equally; 4, many parallel bundles; 5, perfectly parallel bundles). The samples were scored semi-quantitatively: (I) no signs of tissue staining, (II) barely any tissue staining, (III) little staining, (IV) intermediate staining, and (V) strong staining. Examples are given in Supplementary Fig. 1.

### Statistics

Data are presented as mean or median depending on the normality distribution (tested by Shapiro–Wilk test). Data is presented as mean ± standard deviation (SD) or median (IQR) based on the distribution. Group differences were first assessed by one-way ANOVA. If the overall F-test was significant (*p* < 0.05), we applied two post hoc tests: (1) Dunnett’s test to compare each disease group (CKD, PD) with the healthy control reference; (2) Sidak’s test for the remaining CKD versus PD contrast. All data were analyzed using GraphPad Prism 10.2 (GraphPad, CA, USA) and SPSS 29 (IBM, NY, USA). The level of significance was set at 0.05.

### Data availability

The datasets generated and analyzed during the current study are available from the corresponding author on reasonable request. Peritoneal tissue and biofluid samples were obtained from the International Pediatric Peritoneal Biobank (clinicaltrials.gov identifier NCT01893710) and are subject to ethical and legal restrictions.

## Results

### Peritoneal histology

Patient clinical characteristics are summarized in Table 1. All three groups had comparable age and gender distribution. Twenty-two percent of PD patients had experienced peritonitis, and their histological findings were similar to children without peritonitis. In CKD5 and PD patients, peritoneal thickness and microvascular density were higher than in controls. Collagen I/III ratio and collagen fibers alignment did not differ between the groups (Supplementary Fig. 1, Table 2).

**Table 1:** Clinical characteristics of the patient population

	Controls	CKD5	PD	*p*-value (ANOVA)	*p*-value (CKD5 vs. PD)
Gender (f/m)	10/6	6/6	7/10	0.06	0.97
Age (years)	4.6 (0.01, 16.4)	5.25 (0.01, 15.8)	5 (0.01, 14.9)	0.08	0.98
PD duration (months)	n/a	n/a	15 (0.01, 48)	n/a	n/a
Glucose exposure in g/d/BSA	n/a	n/a	450 (55.2, 2550)	n/a	n/a

The cohorts did not differ in gender proportion or age. Glucose exposure was measured in grams per day per body surface area (BSA). Statistics: one-way ANOVA with Sidak´s multiple comparison test as well as Kruskal–Wallis test with Dunn’s post hoc

Next, we quantified the inflammatory cells invading the peritoneal membrane. While the proportion of patients with peritoneal CD45 + leucocytes and CD68 + macrophages was similar, the cell counts were significantly higher in children exposed to PD compared to children without (Table 2).

**Table 2:** Peritoneal tissue histology and markers of neutrophil activation as well as NET formation

	Controls	CKD5	PD	*p*-value (ANOVA)	*p*-value (CKD5 vs. PD)
Submesothelial thickness (µm)	240 (174, 420)	320 (224, 410)	543 (458, 554)	0.002	0.008
Collagen I/III ratio	0 (0, 0)	0 (0, 0.5)	0 (0, 1)	0.84	0.57
Collagen alignment score	0.8 (0.6, 1)	1.33 (0.71, 2)	1.33 (0.7, 2)	0.18	0.92
Microvessel density (/mm^2^)	62 (32, 180)	65 (13, 158)	112 (93, 209)	0.038	0.03
CD45 positive (cells/hpf)	3.4 (3, 5,7)	5 (0, 6)	15 (5, 27)	0.01	0.01
CD68 positive (cells/hpf)	3 (2.7, 5)	2.5 (0, 6)	12 (4, 23)	0.01	0.005
DNase1	1 (1, 1)	2 (1.7, 3)	3 (2, 4)	< 0.001	0.01
DNase1L3	0 (0, 3)	1 (0, 1)	2 (1, 2.5)	< 0.001	0.05
NE	0 (0, 1)	0 (0, 1.5)	1 (0, 2)	0.18	0.30
MPO	0 (0, 0)	0 (0, 1)	0 (0, 1.5)	0.06	0.67
H3cit	0 (0, 1)	0 (0, 1)	1 (0, 2)	0.08	0.20

Parietal peritoneum from 16 children without kidney disease (control), 12 children with chronic kidney disease at the start of peritoneal dialysis (CKD5) and 16 children on peritoneal dialysis (PD) with glucose-based PD fluids with low concentrations of glucose degradation products. Glucose exposure was measured in grams per day per body surface area (BSA). Data are presented as median (IQR). Statistics: one-way ANOVA with Sidak’s multiple comparison test

### NET formation and degradation in peritoneal tissues

**Fig. 1:**
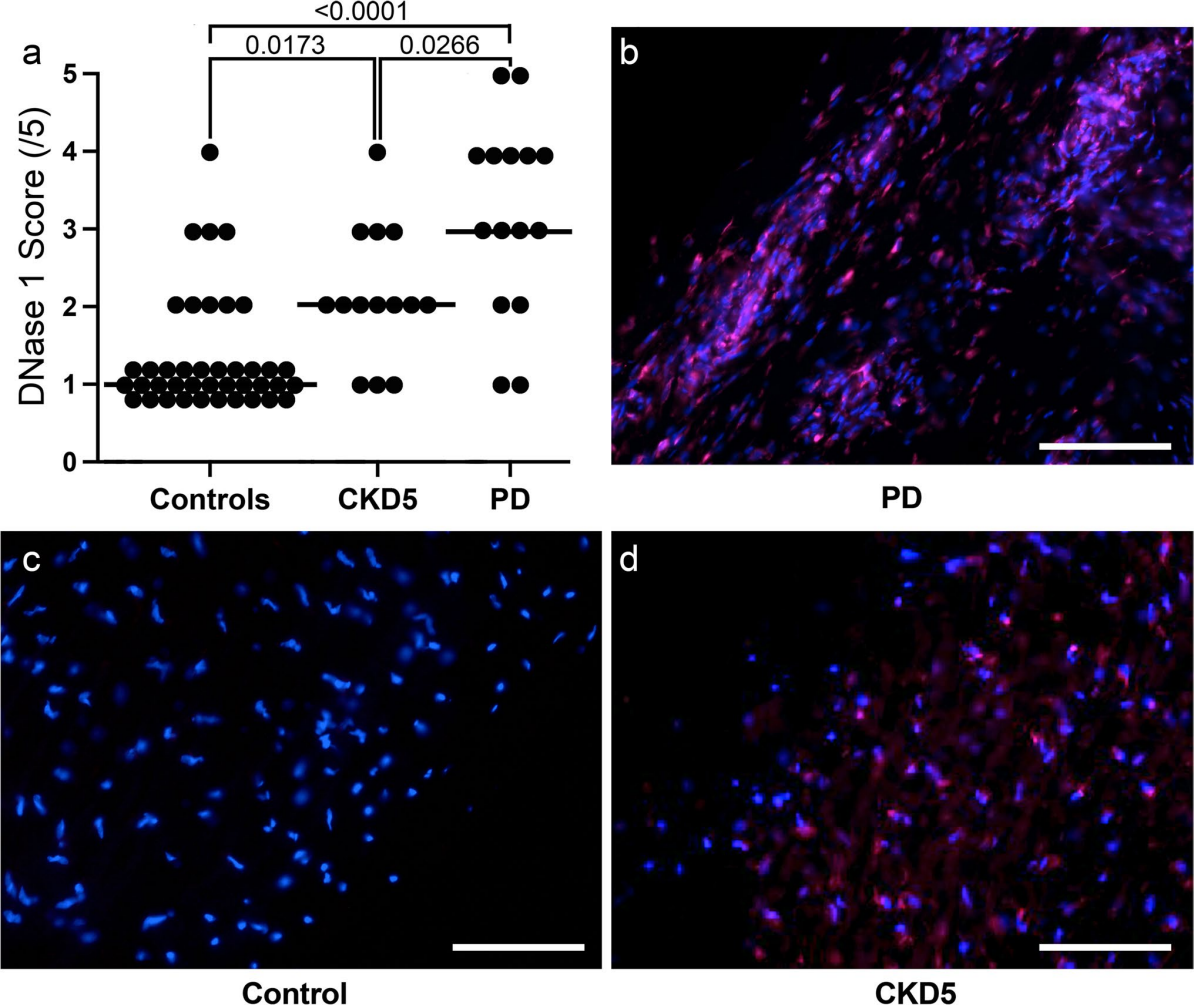
Peritoneal DNAse1 expression rises with kidney disease and PD exposure (**a**). Immunofluorescence staining for DNase1 (red) in peritoneum from children without kidney disease (control) (**c**), children with CKD stage 5 at PD-catheter insertion (CKD5) (**d**), and children on chronic PD (low-GDP solution) at catheter explantation or transplantation (PD) (**b**). Nuclei were counterstained with DAPI (blue). Quantitative DNase1 score was increased; especially in PD patients (one-way ANOVA with Sidák post-test)

**Fig. 2:**
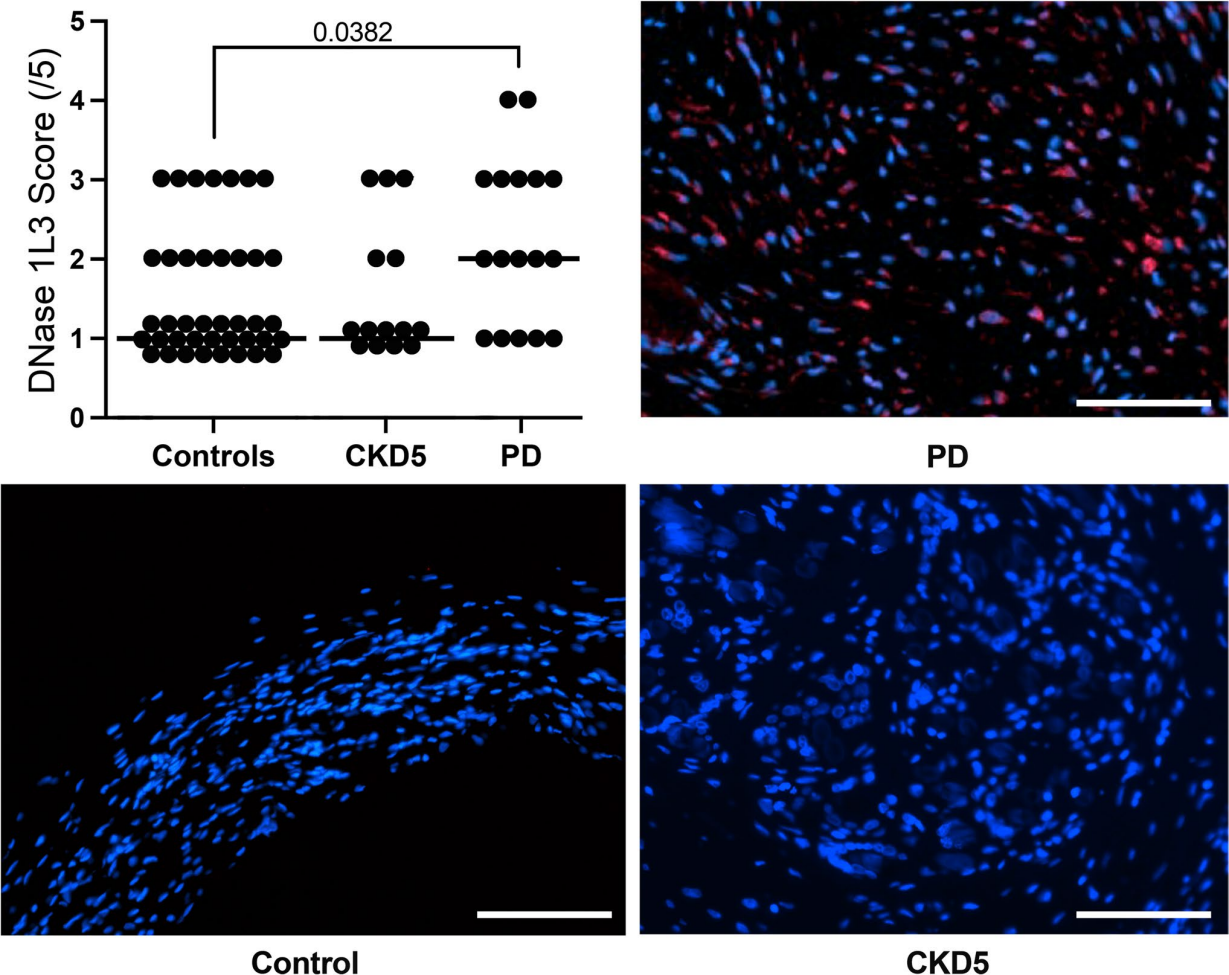
DNase 1-like 3 (DNase1L3) accumulates in the peritoneum during PD. Semi-quantitative histoscores (top left) confirm an elevation in PD patients (one-way ANOVA with Sidák post-test). Immunofluorescence for DNase1L3 (red) with DAPI nuclear counterstain (blue) in control, CKD5, and PD samples. Representative images (left) illustrate progressive intensification of DNase 1L3 signal

**Fig. 3:**
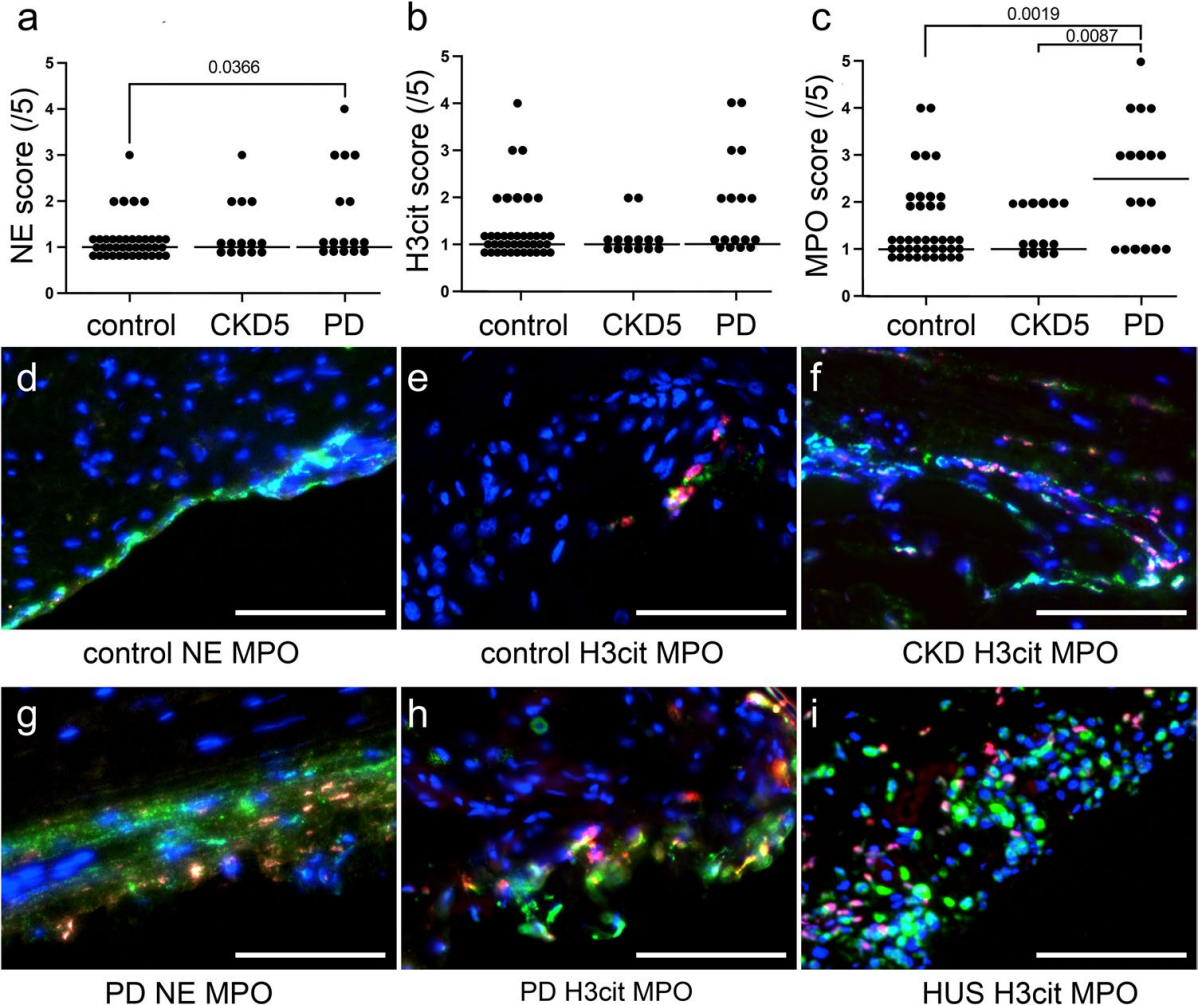
Peritoneal NET signature in PD: up-regulation of NE, citrullinated histone H3 and MPO (**a-c**). MPO was significantly increased especially in PD patients (one-way ANOVA with Sidák post-test). Composite immunofluorescence panels of peritoneal sections from each cohort stained for neutrophil elastase (NE, red) (**d/g**), myeloperoxidase (MPO, green) (**e/h**), and citrullinated histone H3 (H3cit, red) (**f/i**), as well as DAPI (blue)

Peritoneal NET formation was higher in children with CKD5 and PD compared to children without kidney disease (Table 2). The most prominent increase was observed for DNase1 (Fig. 1) and DNase1L3 (Fig. 2), the rate limiting factors of NET accumulation [31]. Neutrophil elastase (NE) score showed higher levels in PD patients when compared to controls but did not differ between children with kidney disease (Fig. 3a). There were no differences for H3cit (Fig. 3b). MPO was higher in patients on PD when compared to CKD5 patients and controls without kidney disease (Fig. 3c).

### Tissue NET activation and degradation markers are associated with the peritoneal tissue transformation

We next analyzed whether the markers for peritoneal inflammation and for formation/degradation of NETs were associated with the PD regimen or peritoneal tissue transformation. Peritoneal levels of DNase1 were associated with those of DNase1L3, NE, H3cit, MPO, and submesothelial thickness (*r* = 0.52/*r* = 0.28/*r* = 0.29/*r* = 0.42/*r* = 0.36, *p* < 0.05 for all); DNase1L3 was associated with peritoneal MPO (*r* = 0.27, *p* = 0.018) and vascular density (*r* = 0.38, *p* = 0.021). Peritoneal levels of the NET markers H3cit, MPO, and macrophage infiltration (CD68 + cells) correlated with the duration of the PD (*r* = 0.28/0.28/0.42, *p* < 0.05 for all). Glucose exposure per day per body surface area was associated with peritoneal abundance of MPO (*r* = 0.66, *p* < 0.001), and with submesothelial thickness (*r* = 0.66, *p* < 0.001). Peritoneal NE was associated with peritoneal H3cit (*r* = 0.40, *p* < 0.001) and MPO (*r* = 0.43, *p* < 0.001), H3cit correlated with peritoneal MPO (*r* = 0.78, *p* < 0.001). Inflammatory cell counts in the peritoneal tissues were also closely related (CD 45 and CD 68 (*r* = 0.65, *p* < 0.001).

### Systemic NET formation and degradation markers are higher in children on PD and associated with inflammation

**Fig. 4:**
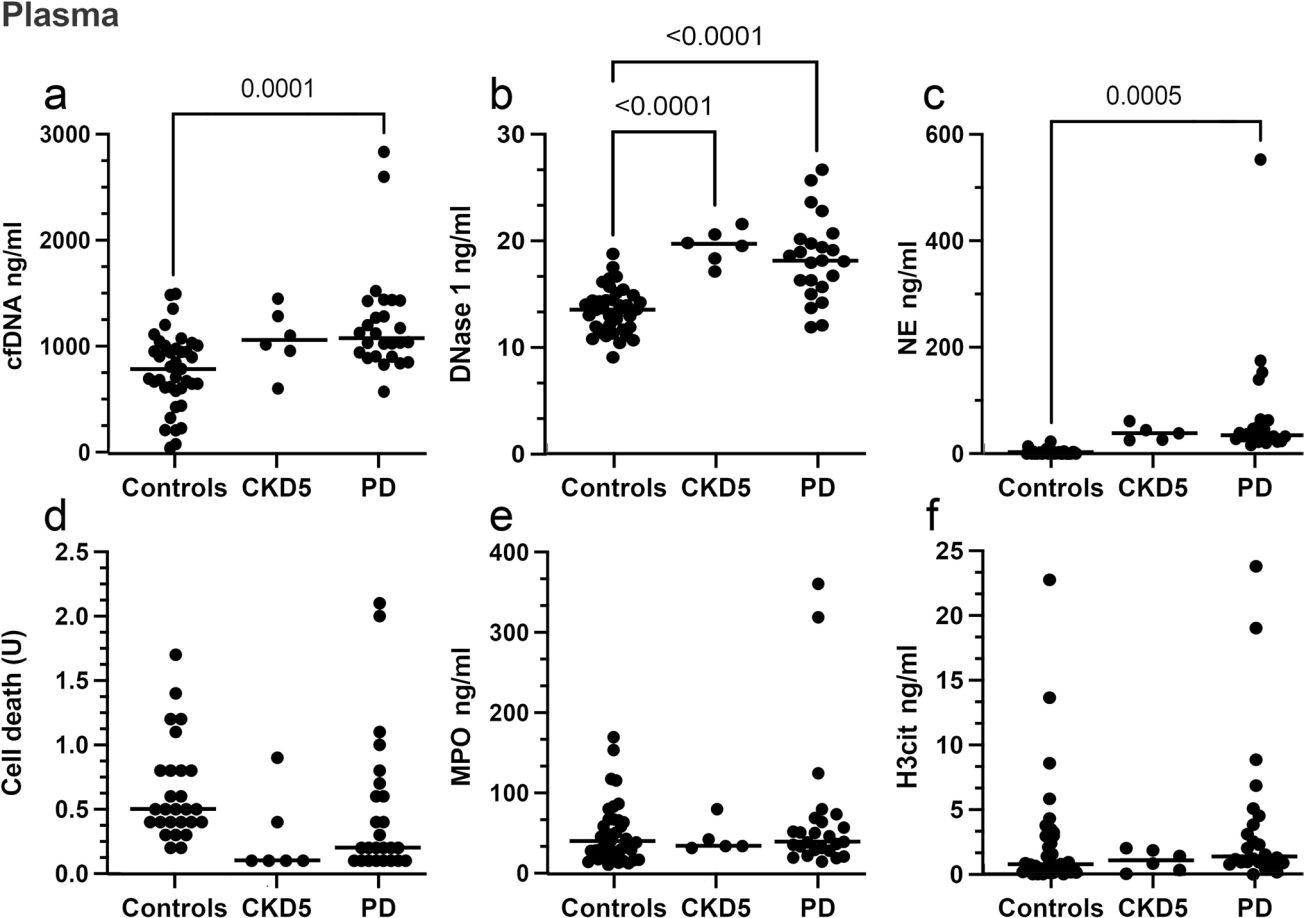
Systemic NET formation and degradation markers are highest in children on PD. Plasma concentrations of cell-free DNA (cfDNA), NE, MPO, H3cit, DNase 1 in controls, CKD5 and PD patients. Systemically cfDNA (**a**) and NE (**c**, NET formation), and DNase1 (**b**, NET degradation) were elevated in PD patients. Group differences were analyzed with one-way ANOVA followed by Sidák correction

Next to the local tissue abundance, the systemic levels of NET markers were quantified in plasma of CKD5 and PD patients and in dialysate from PD patients. Plasma concentrations of cell free (cf) DNA, DNAse1, and NE were the highest in children on PD compared to children without kidney disease (Fig. 4). PD duration was associated with plasma DNase1 (*r* = −0.38, *p* = 0.001); cfDNA (*r* = 0.35, *p* = 0.002); NE (*r* = 0.28, *p* = 0.03); H3cit (*r* = −0.25, *p* = 0.03); and cell death (*r* = −0.43, *p* < 0.001).

In contrast, plasma levels of MPO, H3cit, and cell death markers did not differ between the cohorts, but were still associated with PD regimen and other NET markers, especially those for NET degradation. H3cit in plasma correlated with PD duration (*r* = 0.48, *p* < 0.001); plasma DNase1 (*r* = 0.39, *p* = 0.001); cfDNA (*r* = −0.42, *p* < 0.001); NE (*r* = −0.24, *p* < 0.001); and cell death (*r* = 0.59, *p* < 0.001) as well as levels of DNase1 (*r* = −0.28, *p* = 0,04) and CD68 (*r* = 0.46, *p* = 0.037) in peritoneal tissue. The markers for NET formation and degradation in plasma were closely associated, and DNase1 was associated with cfDNA (*r* = −0.56, *p* < 0.001); NE (*r* = −0.38, *p* = 0.001); H3cit (*r* = 0.39, *p* = 0.001); MPO (*r* = −0.28, *p* = 0.031); cell death (*r* = 0.78, *p* < 0.001) and cfDNA was correlated with blood NE (*r* = 0.53, *p* < 0.001); MPO (*r* = 0.46, *p* < 0.001); and cell death (*r* = −0.65, *p* < 0.001). Blood NE correlates with blood MPO (*r* = 0.92, *p* < 0.001); cell death (*r* = −0.41, *p* < 0.001); and peritoneal abundance of DNase1 (*r* = 0.28, *p* = 0.039).

**Fig. 5:**
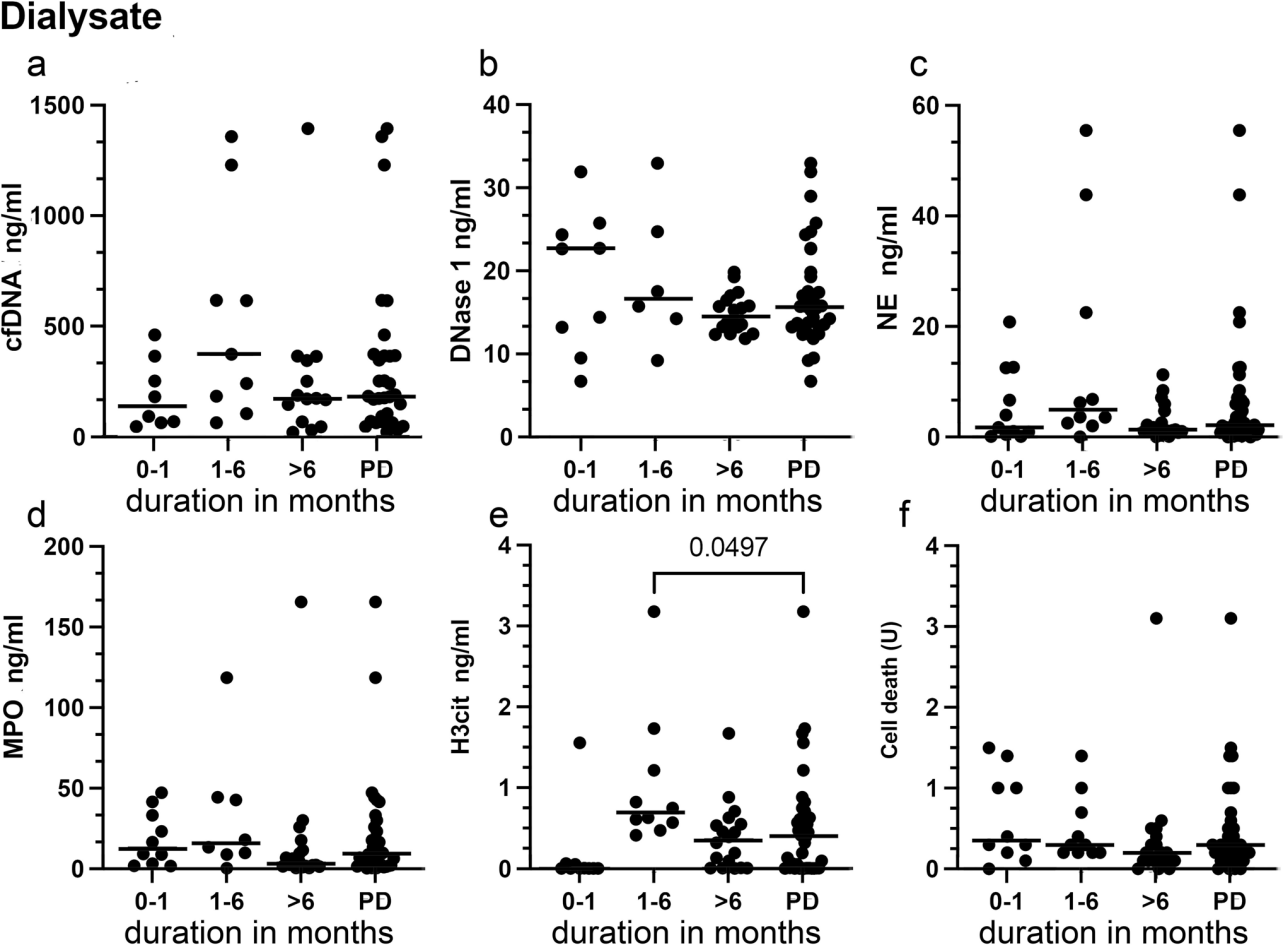
Systemic NET formation and degradation markers are detectable in dialysate in children on PD. Dialysate concentrations of cell-free DNA (cfDNA), NE, MPO, H3cit, DNase 1 in controls, CKD5 and PD patients. H3cit levels in dialysate were elevated in patients in the early months of PD (first to sixth month) compared to the full cohort of PD patients included in our study (e). Group differences were analyzed with one-way ANOVA followed by Sidák correction

The same markers were also quantified in the dialysate samples from CKD 5 patients after PD initiation and on stable PD (Fig. 5). We observed no time-dependency and the longitudinal measurements of the dialysate from the same patients showed stable values. Furthermore, no associations were observed for the duration of the PD and dialytic NET markers. Dialysate concentration of H3cit correlated with NE (*r* = 0.67, *p* < 0.001) and dialysate NE with MPO (*r* = 0.97, *p* < 0.001).

The heatmap in Supplement Fig. 2 displays Spearman correlation coefficients (*r*-values) between plasma markers of NET formation (cfDNA, NE, MPO, cell-death ELISA) and degradation (DNase1) pooled from CKD5 and PD samples.

## Discussion

The current study provides novel insights into the inflammatory mechanisms underlying peritoneal membrane alterations in pediatric patients undergoing peritoneal dialysis (PD). The data illustrate that low glucose degradation product (GDP) PD fluids, despite their advantages over high GDP solutions, still induce significant inflammatory responses. These are characterized by increased formation of neutrophil extracellular traps (NETs). Elevated local and systemic NET markers such as neutrophil elastase (NE), myeloperoxidase (MPO), citrullinated histone H3 (H3cit), and cell-free DNA (cfDNA), as well as NET degradation enzymes (DNase1 and DNase1L3), were strongly correlated with histological indicators of peritoneal remodeling. These include increased membrane thickness, enhanced vascularization, and macrophage infiltration. Importantly, we now demonstrate that these NET markers retain independent associations even after adjustment for residual glomerular filtration rate (eGFR), underscoring that systemic NET accumulation cannot be explained by impaired renal clearance alone.

Importantly, systemic NET markers also correlated significantly with the clinical parameters glucose exposure and duration of PD therapy. The robust correlations observed among these markers suggest a coordinated inflammatory process, underscoring the complexity and extent of the immune response during PD. Systemic NET markers also correlated with dialysate glucose load and PD vintage, suggesting a chronic, glucose-driven inflammatory circuit.

These findings align with previous observations that chronic exposure to glucose-rich dialysis fluids drives inflammation-mediated tissue remodeling [2]. While glucose degradation products have been largely removed form PD solutions used in the majority of European countries [32], currently used peritoneal dialysis fluids are still far from biocompatible, containing high supraphysiological concentrations of glucose. Glucose affects the peritoneal membrane and induces pathophysiological immune responses that result in progressive transformation of peritoneal membrane [2].

Neutrophil influx and associated NETs formation are the first responses to peritoneal inflammation and infection [27, 33]. Chemical gradient is created by chemoattractants of bacterial origin or in case of sterile inflammation by C-X-C motif-containing chemokines and results in infiltration of the peritoneal membrane by neutrophils. NET formation is a process of releasing the decondensed nuclear DNA which forms a net of aggregated neutrophils and can entrap the microorganism. The role of NETs formation in sterile inflammation is less well described although they entrap many kinds of sterile particulate matter [34–38].

During the NET formation, production of IL-1b, IL-6, IL-8, IL-18, and TNFα from macrophages is induced, activates the NLRP3 inflammasome, and promotes inflammation. NETs directly induce monocyte activation and prime CD4 + T-cells. In a feedback loop mechanism, activated neutrophils produce NETs, and these activate neutrophils either directly through the secretion of cytokines, by oxidative stress or IL-1b/IL-18 [39]. The downstream consequences may extend beyond local inflammation: NET-derived histones and DNA-histone complexes can enter the circulation, drive endothelial dysfunction, and promote pro-thrombotic states, all of which are relevant for the long-term cardiovascular morbidity observed in pediatric CKD.

The degradation of NETs is mediated by DNases, which cleave extracellular DNA, including NETs, into small DNA protein aggregates. The most abundant extracellular deoxyribonucleases are members of the DNase1 protein family. DNase1 is expressed by nonhematopoietic tissues and preferentially cleaves protein-free DNA [23]. DNase1L3 is secreted by immune cells and additionally targets DNA–protein complexes, such as nucleosomes. DNases have anti-inflammatory effects either via interaction of NETs and platelets or via disruption of the self-amplifying loop between activated neutrophils and NETs.

We observed an abundance of DNase1 in tissues as well as in plasma, suggesting that not only NET activation but also NET degradation pathways are operating. In 30 adult hemodialysis patients, Bieber et al. [40] found increased plasma markers of neutrophil activation after the dialysis session compared to the pre-dialysis stage. These were associated with sICAM1, an important marker of endothelial activation. In HD, the blood is in contact with the external dialyzer that initiates immune responses and activates the endothelia [41]. In contrast, in patients on PD, their own peritoneum serves as dialyzer. Consistent with this paradigm, our PD cohort exhibited intermediate NET levels—higher than CKD5 but lower than published HD cohorts—supporting a continuum of neutrophil activation proportional to extracorporeal exposure.

Ultimately, kidney function may affect NET formation indirectly. Intact NETs are not subject to kidney elimination. However, tubular epithelium is a major source of circulating DNase I; once fragments are small enough, they are filtered and partly excreted. Recently, it has been shown that residual GFR and intrarenal DNase activity shape systemic NET burden [42].

Thus, declining residual GFR could contribute to NET accumulation. However, in our cohort adjustment for eGFR only modestly attenuated NET–remodeling associations, indicating additional drivers such as intraperitoneal NET formation.

## Limitations and outlook

Our studies are limited by the number of available peritoneal samples from children. Although the number of patients per group were rather small, we analyzed tissues from children who mostly suffer from CAKUT without inflammatory disease, allowing for a specific analysis. We obtained corresponding samples of plasma and dialysate of CKD5 and PD patients, yet we did not measure urinary NET fragments, complement activation products, or platelet-NET aggregates. We do not provide mechanistic insights on NET activation; in the future our results will be further validated in a preclinical model using genetic mice for DNase1 or DNase1L3 treated with PD. Future work should employ chronic PD mouse models, including DNase1- or PAD4-deficient strains, to dissect causality and test enzyme supplementation.

Clinically, our data emphasize the importance of monitoring inflammation as a potential indicator of membrane integrity and PD efficacy. Therapeutically, targeting NET formation or enhancing NET degradation pathways may offer novel strategies to mitigate peritoneal damage, preserve residual kidney function, and ultimately improve outcomes in pediatric patients.

In conclusion, NET formation may represent a significant pathogenic factor that contributes to peritoneal membrane damage in pediatric PD patients. Therapeutic interventions aimed at modulating NET activity hold promise for reducing peritoneal inflammation, maintaining membrane functionality, and enhancing long-term patient prognosis. NET formation and insufficient degradation appear to be central, modifiable drivers of peritoneal membrane damage during pediatric PD. Interventions that neutralize or clear NETs, alongside further refinement of biocompatible PD solutions, hold promise to safeguard membrane function and improve long-term outcomes.

## Supplementary Information

Below is the link to the electronic supplementary material.

Graphical abstract (PPTX 77 KB)

Supplementary file2 Collagen architecture of the parietal peritoneum is preserved under PD despite membrane thickening. Picro-Sirius-red–stained sections viewed under polarized light illustrate collagen type I (orange-red birefringence) and type III (yellow-green birefringence) fibers in (I) Controls, (II) children with CKD5 at PD-catheter insertion, and (III) children on chronic PD (low-GDP solution). Representative high-power fields are accompanied by plots of the semi-quantitative collagen-I/III ratio and alignment score (1 = random orientation; 5 = perfectly parallel bundles). Although overall sub-mesothelial thickness increased from Control → CKD5 → PD, neither the relative proportion of collagen I to III nor fiber orientation differed significantly among groups (one-way ANOVA with Sidák post-test) (JPG 3588 KB)

Supplementary file3 Tight inter-relations among circulating NET markers in CKD5/PD. Heatmap displaying Spearman correlation coefficients (r-values) between plasma markers of NET formation (cfDNA, NE, MPO, cell-death ELISA) and degradation (DNase 1) pooled from CKD5 and PD samples. Positive correlations are shown in red, negative in green; blank squares denote p ≥ 0.05. The strong positive clustering of cfDNA/NE/MPO and inverse relation to DNase1 highlight a coordinated systemic NET response (JPG 24 KB)
