# Supplementary material for: Neutrophil extracellular traps drive peritoneal inflammation and tissue remodeling in pediatric peritoneal dialysis
Source: Pediatr Nephrol. 2025 Oct 18;41(3):819–29. doi: 10.1007/s00467-025-07003-w (PMC12852172; doi:10.1007/s00467-025-07003-w)
Supplement: Supplementary file 1 — Graphical abstract (PPTX 77 KB) [file 467_2025_7003_MOESM1_ESM.pptx]

## Slide 1
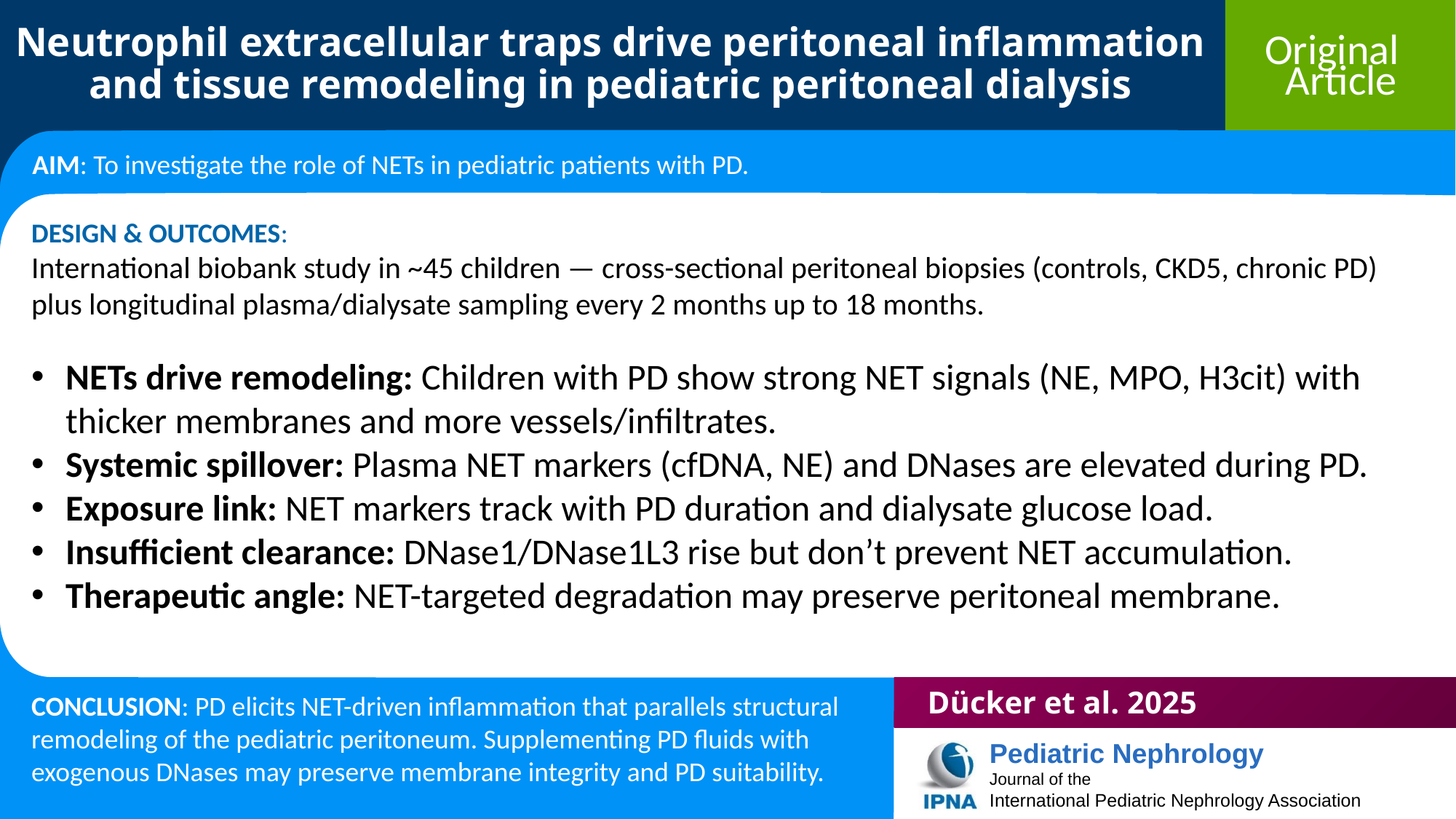

Neutrophil extracellular traps drive peritoneal inflammation and tissue remodeling in pediatric peritoneal dialysis
AIM: To investigate the role of NETs in pediatric patients with PD.
DESIGN & OUTCOMES:
International biobank study in ~45 children — cross-sectional peritoneal biopsies (controls, CKD5, chronic PD) plus longitudinal plasma/dialysate sampling every 2 months up to 18 months.
NETs drive remodeling: Children with PD show strong NET signals (NE, MPO, H3cit) with thicker membranes and more vessels/infiltrates.
Systemic spillover: Plasma NET markers (cfDNA, NE) and DNases are elevated during PD.
Exposure link: NET markers track with PD duration and dialysate glucose load.
Insufficient clearance: DNase1/DNase1L3 rise but don’t prevent NET accumulation.
Therapeutic angle: NET-targeted degradation may preserve peritoneal membrane.
Dücker et al. 2025
CONCLUSION: PD elicits NET-driven inflammation that parallels structural remodeling of the pediatric peritoneum. Supplementing PD fluids with exogenous DNases may preserve membrane integrity and PD suitability.
