# Supplementary figures and images for: Neutrophil extracellular traps drive peritoneal inflammation and tissue remodeling in pediatric peritoneal dialysis
Source: Pediatr Nephrol. 2025 Oct 18;41(3):819–29. doi: 10.1007/s00467-025-07003-w (PMC12852172; doi:10.1007/s00467-025-07003-w)

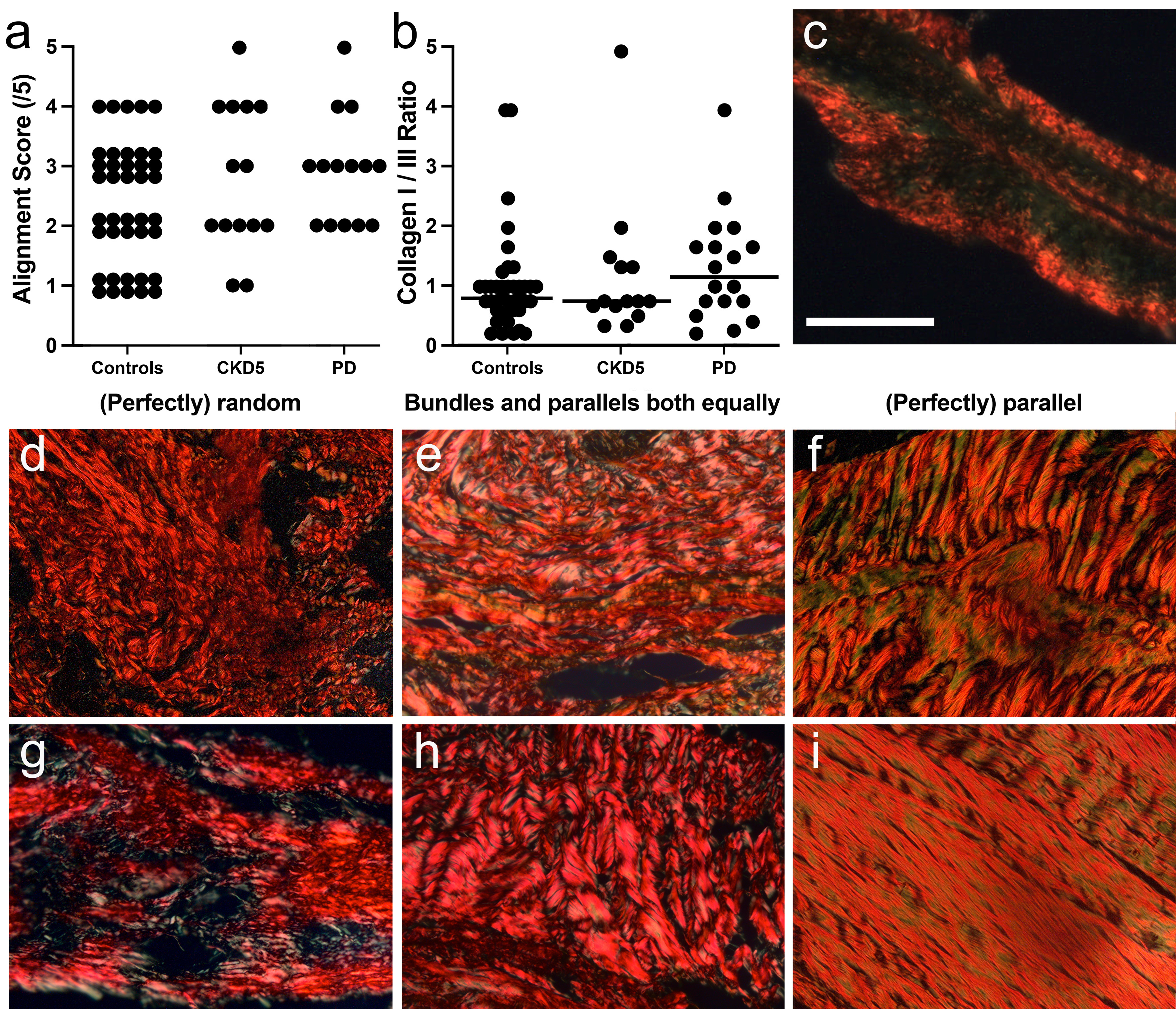

Supplement: Supplementary file 2 — Supplementary file2 Collagen architecture of the parietal peritoneum is preserved under PD despite membrane thickening. Picro-Sirius-red–stained sections viewed under polarized light illustrate collagen type I (orange-red birefringence) and type III (yellow-green birefringence) fibers in (I) Controls, (II) children with CKD5 at PD-catheter insertion, and (III) children on chronic PD (low-GDP solution). Representative high-power fields are accompanied by plots of the semi-quantitative collagen-I/III ratio and alignment score (1 = random orientation; 5 = perfectly parallel bundles). Although overall sub-mesothelial thickness increased from Control → CKD5 → PD, neither the relative proportion of collagen I to III nor fiber orientation differed significantly among groups (one-way ANOVA with Sidák post-test) (JPG 3588 KB) [file 467_2025_7003_MOESM2_ESM.jpg]

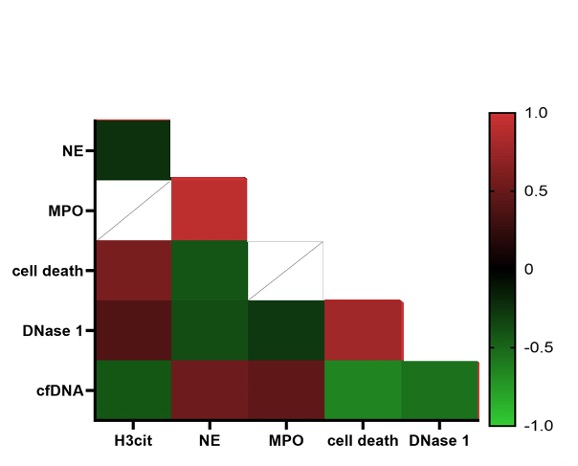

Supplement: Supplementary file 3 — Supplementary file3 Tight inter-relations among circulating NET markers in CKD5/PD. Heatmap displaying Spearman correlation coefficients (r-values) between plasma markers of NET formation (cfDNA, NE, MPO, cell-death ELISA) and degradation (DNase 1) pooled from CKD5 and PD samples. Positive correlations are shown in red, negative in green; blank squares denote p ≥ 0.05. The strong positive clustering of cfDNA/NE/MPO and inverse relation to DNase1 highlight a coordinated systemic NET response (JPG 24 KB) [file 467_2025_7003_MOESM3_ESM.jpg]
